# *CETP*, *LIPC*, and *SCARB1* variants in individuals with extremely high high-density lipoprotein-cholesterol levels

**DOI:** 10.1038/s41598-019-47456-2

**Published:** 2019-07-29

**Authors:** Chan Joo Lee, Mun Su Park, Miso Kim, Soo-jin Ann, Jaeho Lee, Sungha Park, Seok-Min Kang, Yangsoo Jang, Ji Hyun Lee, Sang-Hak Lee

**Affiliations:** 1 Division of Cardiology, Department of Internal Medicine, Severance Hospital, Yonsei University College of Medicine, Seoul, Korea; 2 Department of Biomedical Science and Technology, Graduate School, Kyung Hee University, Seoul, Korea; 3 Cardiovascular Research Institute, Yonsei University College of Medicine, Seoul, Korea; 4 Department of Clinical Pharmacology and Therapeutics, College of Medicine, Kyung Hee University, Seoul, Korea; 5 Department of Biomedical Science and Technology, Kyung Hee Medical Science Research Institute, Kyung Hee University, Seoul, Korea

**Keywords:** Metabolic disorders, Cardiovascular genetics, Clinical genetics

## Abstract

The concentration of high-density lipoprotein-cholesterol (HDL-C) in humans is partially determined by genetic factors; however, the role of these factors is incompletely understood. The aim of this study was to examine the prevalence and characteristics of *CETP*, *LIPC*, and *SCARB1* variants in Korean individuals with extremely high HDL-C levels. We also analysed associations between these variants and cholesterol efflux capacity (CEC), reactive oxygen species (ROS) generation, and vascular cell adhesion molecule-1 (VCAM-1) expression. Of 13,545 participants in the cardiovascular genome cohort, 42 subjects with HDL-C levels >100 mg/dL were analysed. The three target genes were sequenced by targeted next-generation sequencing, the functional effects of detected variants were predicted, and CEC was assessed using a radioisotope and apolipoprotein B-depleted sera. We observed two rare variants of *CETP* in 13 individuals (rare variant c.A1196G [p.D399G] of *CETP* was discovered in 12 subjects) and one rare variant of *SCARB1* in one individual. Furthermore, all subjects had at least one of four common variants (one *CETP* and three *LIPC* variants). Two additional novel *CETP* variants of unknown frequency were found in two subjects. However, the identified variants did not show significant associations with CEC, ROS generation, or VCAM-1 expression. Our study provides additional insights into the role of genetics in individuals with extremely high HDL-C.

## Introduction

Plasma concentrations of cholesterol are generally known to be influenced by the combined effects of diverse genetic variants^1^. Individuals with extremely high lipid levels have a greater chance of being affected by monogenic syndrome in comparison to those with lipid levels closer to the normal values^2–4^. In addition, research into lipid phenotypes has played a key role in highlighting therapeutic targets^5^.

While the concentration of the high-density lipoprotein-cholesterol (HDL-C) is known to be partly determined by genetic factors, these factors are not entirely clear yet. In this regard, genetic variants associated with extremely high levels of HDL-C are under steady investigation^6^, and it has been shown that more than 40 genes are associated with HDL-C levels^7^. Interestingly, a recent large study involving a combination of multiple consortia showed that truncation variants of *CETP* were associated with high HDL-C levels and a low risk of coronary artery disease^8^. Although several genetic studies on HDL-C have been performed to date in Japan^9,10^, comprehensive studies on genetic variants that influence HDL-C in Asians are still limited.

The aim of our study was to investigate the prevalence and characteristics of *CETP*, *LIPC*, and *SCARB1* variants in Korean individuals with extremely high levels of HDL-C using next-generation sequencing, as rare variants of these genes are known to be associated with the above-mentioned HDL-C phenotype^2,11^. Additionally, we analysed associations between the identified variants and individuals’ cholesterol efflux capacity (CEC), a functional parameter of HDL that can be affected by metabolic changes in variant carriers.

## Results

### Clinical characteristics of study subjects

The mean age of 42 study subjects was 54 years, of which 16 (38%) were males. Two subjects (5%) had diabetes mellitus, and three (7%) had coronary artery disease. The mean HDL-C level was 110.1 ± 12.6 mg/dL. In comparison to the characteristics of the total cohort population, the study subjects were younger, had a lower prevalence of cardiovascular risk factors, and had lower triglyceride levels (Table 1).

**Table 1:** Clinical characteristics of study subjects.

	Total population	Subjects with very high HDL-C	*p*
	(n = 13545)	(n = 42)	
Age, years	60.4 ± 10.6	54.1 ± 12.8	<0.0001
Male	6722 (50)	16 (38)	0.12
**Medical history**			
Hypertension	7234 (53)	9 (21)	<0.0001
Diabetes mellitus	2293 (17)	2 (5)	<0.0001
Smoking	2004 (15)	11 (26)	0.09
Coronary artery disease	4741 (35)	3 (7)	<0.0001
Body mass index, kg/m^2^	24.8 ± 3.1	22.6 ± 4.2	0.002
**Laboratory values, mg/dL**			
Total cholesterol	189 ± 43	220 ± 38	<0.0001
TG	117 (83)	62.5 (27)	<0.0001
HDL-C	48.8 ± 14.7	110.1 ± 12.6	<0.0001
LDL-C	115 ± 38	101 ± 35	0.02

Data are presented as mean ± standard deviation or number (%); HDL-C: high-density lipoprotein-cholesterol; TG: triglyceride; LDL-C: low-density lipoprotein-cholesterol.

### Analysis of candidate genes

**Figure 1:**
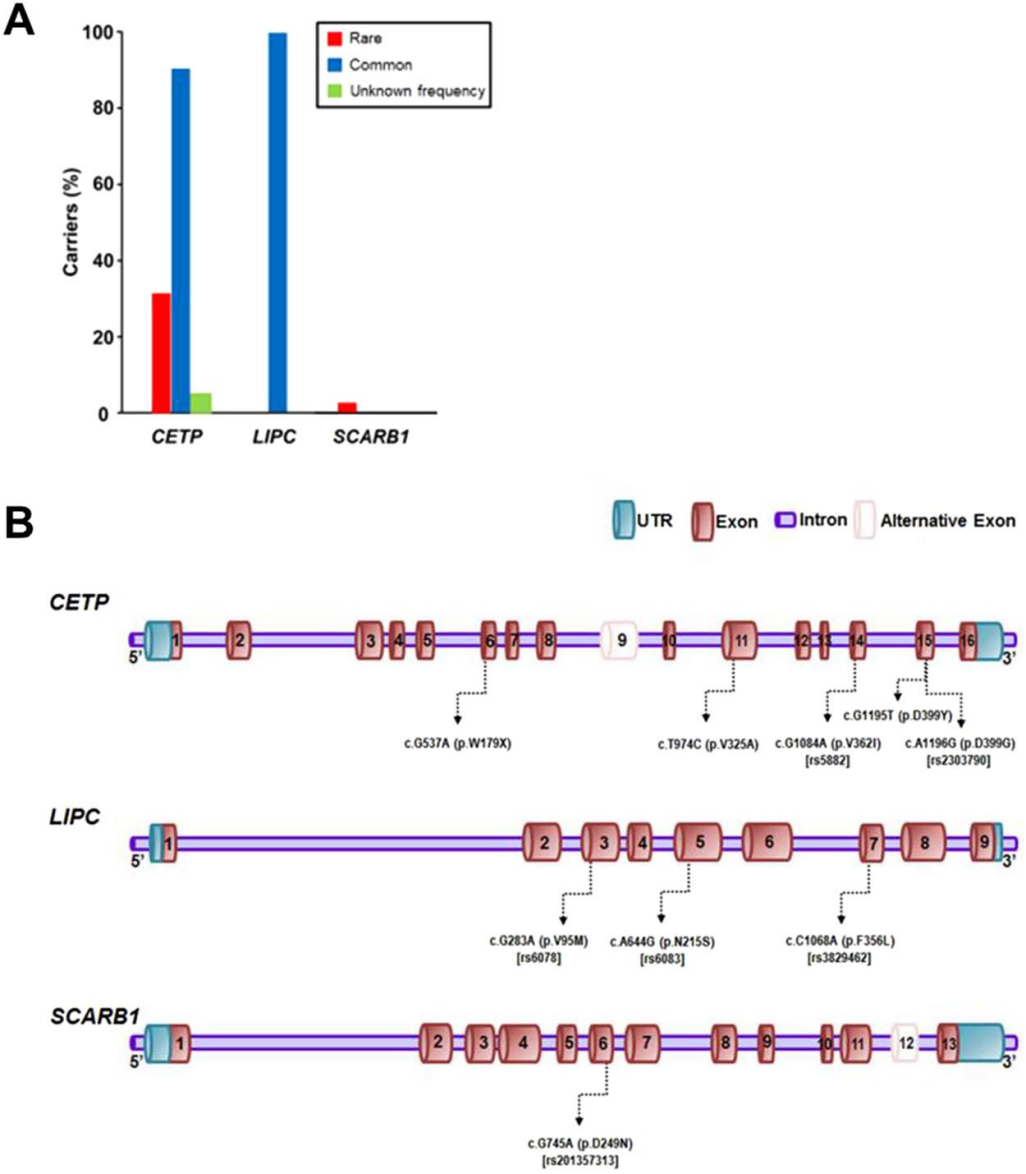
Proportion of variant carriers and locations of variants in each gene. (**A**) Proportion of carriers who had variants in each of the three genes identified in the 42 study subjects. With regard to *CETP*, 13 subjects carried rare variants, and 38 subjects had common variants. Two variants of unknown frequency in *CETP* were discovered in two individuals. No rare variant was identified, while common variants of *LIPC* were found in all subjects. Conversely, with regard to *SCARB1*, one rare variant was discovered in one subject. (**B**) Locations of *CETP*, *LIPC*, and *SCARB1* variants identified in the study subjects.

In the 42 subjects, rare and common variants of *CETP* and common variants of *LIPC* were frequent. Two rare variants of *CETP* were present in 13 individuals (31%), whereas one rare variant of *SCARB1* was observed in one subject (Table 2). In particular, a rare variant of *CETP* (c.A1196G [p.D399G]) was discovered in 12 subjects (29%) and the rate was much higher than that in the gnomAD database (Fig. 1A). Conversely, all 42 study subjects had at least one of four common variants of *CETP* or *LIPC*. One common variant of *CETP* (c.G1084A [p.V362I]) and two common variants of *LIPC* (c.C1068A [p.F356L] and c.A644G [p.N215S]) were highly frequent, and were found in 38, 42, and 42 subjects, respectively (Table 2, Fig. 1A). A summary of the genetic variants found in each individual is presented in Supplementary Table S1, and the locations of the variants in each of the three genes are indicated in Fig. 1B.

**Table 2:** Genetic variants identified in target genes in study subjects.

Gene	Genomic coordinate	Nucleotide change*	Mutation type	Amino acid change (rs number in dbSNP)	Allele frequency	Frequency in gnomAD database	Affected patients (homo-/heterozygous)	Reported**	SIFT/Polyphen/Mutation- Taster prediction (Clinical significance based on clinVar)
*CETP*		Rare							
	chr16: 57,017,292	c.A1196G	nonsynonymous SNV	p.D399G (rs2303790)	12 (0.286)	0.0000–0.03281	1/11	Yes	Deleterious/possibly damaging/polymorphism
	chr16: 57,017,291	c.G1195T	nonsynonymous SNV	p.D399Y	1 (0.024)	NA	0/1	Yes	Deleterious/probably damaging/polymorphism
		Common							
	chr16: 57,016,092	c.G1084A	nonsynonymous SNV	p.V362I (rs5882)	38 (0.881)	0.4194–0.6197	15/23	Yes	Tolerated/benign/polymorphism_automatic
		Unknown frequency							
	chr16: 57,015,077	c.T974C	nonsynonymous SNV	p.V325A	1 (0.024)	NA	0/1	No	Tolerated/benign/polymorphism
	chr16: 57,004,954	c.G537A	stop-gain SNV	p.W179X	1 (0.024)	NA	0/1	No	
*LIPC*		Common							
	chr15: 58,853,079	c.C1068A	nonsynonymous SNV	p.F356L (rs3829462)	42 (1.000)	0.8570–0.9969	32/10	Yes	Tolerated/benign/polymorphism_automatic
	chr15: 58,838,010	c.A644G	nonsynonymous SNV	p.N215S (rs6083)	42 (1.000)	0.3260–0.8322	30/12	Yes	Deleterious/benign/polymorphism_automatic
	chr15: 58,833,993	c.G283A	nonsynonymous SNV	p.V95M (rs6078)	16 (0.381)	0.01886–0.3325	0/16	Yes	Tolerated/benign/polymorphism_automatic
*SCARB1*		Rare							
	Chr12: 125,294,817	c.G745A	nonsynonymous SNV	p.D249N (rs201357313)	1 (0.024)	NA	0/1	Yes	

SNV: single nucleotide variant, NA: not available, *Nucleotide location number was assigned according to the *CETP* (NM_001286085), *LIPC* (NM_000236), and *SCARB1* (NM_005505) mRNA sequences. **Reported in gnomAD browser.

### CETP

Two rare variants of *CETP* were found in 13 subjects: c.A1196G (p.D399G) in 12 individuals and c.G1195T (p.D399Y) in one individual. One carrier of c.A1196G (p.D399G) was homozygous for this variant, while the others were heterozygous. All rare variants of this gene observed in the present study have been previously reported and were predicted to be damaging. Conversely, one common variant, c.G1084A (p.V362I), was identified in 38 subjects, of which 15 carriers were homozygous for this variant. This common variant was suspected to be benign according to the prediction programs used here. Furthermore, two novel variants of unknown frequency—c.T974C (p.V325A) and c.G537A (p.W179X)—were discovered in two individuals (Table 2).

### LIPC

No rare variants of *LIPC* were identified, and three common variants of *LIPC* were found among the study subjects: c.C1068A (p.F356L), c.A655G (p.N215S), and c.G283A (p.V95M) in 42, 42, and 16 individuals, respectively. In most cases, the carriers of the first two variants were homozygous, whereas all carriers of the last variant were heterozygous. These three variants have also been previously reported. Of the variants, only c.A655G (p.N215S) was predicted to be disease-causing, whereas the others were suspected to be tolerated (Table 2).

### SCARB1

One rare variant of *SCARB1* was discovered in one subject: c.G745A (p.D249N). The carrier was heterozygous for this previously reported variant. According to *in silico* analyses, the effect of this variant was predicted to be uncertain. No common variants of *SCARB1* were found in our study subjects (Table 2).

### Associations of the variants with CEC, reactive oxygen species (ROS), and vascular cell adhesion molecule-1 (VCAM-1)

When subjects were categorized according to the quartile CEC values, there were no significant differences in the numbers of variants of all target genes or of *CETP* or *LIPC* alone. Owing to statistical limitations, the relationship of the *SCARB1* variant with CEC could not be analysed. In addition, the numbers of variant carriers were compared between the four groups of subjects as categorized by quartile CEC values. For each of the five variants of *CETP*, three variants of *LIPC*, and one variant of *SCARB1*, there were no differences in the number of carriers between the four quartile groups (Table 3). CEC values were compared between the carriers and non-carriers of three variants, c.A1196G (p.D399G) and c.G1084A (p.V362I) in *CETP*, and c.G283A (p.V95M) in *LIPC*. However, the values were similar between the carriers and non-carriers of these three variants (Supplementary Table S4). For other variants, it was not statistically appropriate to compare carriers and non-carriers because the sample sizes were too small.

**Table 3:** Association between the burden of variants and individual’s cholesterol efflux capacity.

	Quartiles of cholesterol efflux capacity				*p*
	1^st^ (n = 11)	2^nd^ (n = 10)	3^rd^ (n = 10)	4^th^ (n = 11)	
Cholesterol efflux capacity	17.2 (16.4–18.1)	22.5 (22.1–23.1)	25.7 (25.2–26.1)	31.3 (29.1–33.0)	<0.001
**Numbers of variants in subjects of each quartile group**					
All target genes	3 (2.5–3)	4 (3–5)	4 (3–4)	4 (2.5–4)	0.26
*CETP*	1 (1–1.5)	1 (1–2)	1 (1–2)	1 (1–2)	0.95
*LIPC*	2 (1–2)	2.5 (2–3)	2 (2–2)	2 (2–2.5)	0.051
**Numbers of variant carriers in each quartile group**					
*CETP*					
c.A1196G (p.D399G)	2	3	4	2	0.62
c.G1195T (p.D399Y)	0	0	0	1	0.41
c.G1084A (p.V362I)	10	9	9	9	0.90
c.T974C (p.V325A)	0	0	0	1	0.41
c.G537A (p.W179X)	1	0	0	0	0.41
*LIPC*					
c.C1068A (p.F356L)	6	9	8	10	0.14
c.A644G (p.N215S)	10	10	10	9	0.52
c.G283A (p.V95M)	3	6	3	5	0.40
*SCARB1*					
c.G745A (p.D249N)	0	1	0	0	0.35

Data are presented as median (interquartile range) or number.

Likewise, when subjects were categorized according to the quartile ROS generation, there was no significant difference in the numbers of variants of all target genes or of *CETP* or *LIPC* alone. For each of variant of three target genes, there were no differences in the number of carriers between the four quartile groups (Supplementary Table S2). ROS generation were similar between the carriers and non-carriers of c.A1196G and c.G1084A in *CETP*, and c.G283A in *LIPC* (Supplementary Table S4).

When subjects were categorized according to the quartile VCAM-1 expression, there was no significant difference in the numbers of variants of all target genes or of *CETP* or *LIPC* alone. For each of variant of three target genes, there were no differences in the number of carriers between the four quartile groups. However, the number of carriers of c.A1196G in *CETP* tended to be higher in lower quartile group of VCAM-1 expression (Supplementary Table S3). Although VCAM-1 expression were not significantly different between the carriers and non-carriers of c.A1196G and c.G1084A in *CETP*, and c.G283A in *LIPC*, the carriers of c.A1196G showed tendency of lower VCAM-1 expression (Supplementary Table S4).

## Discussion

In our study population with extremely high HDL-C levels, rare or common variants of *CETP* and common variants of *LIPC* were frequent. All the study subjects were carriers of more than one common variant of *CETP* or *LIPC*. Only one individual possessed a *SCARB1* variant. All rare or common variants had been previously reported, while two novel *CETP* variants of unknown frequency were observed in two individuals. In our study population, we did not identify any significant associations between the identified variants and the CEC values of the subjects. These results provide rare and informative data on the genetic spectra of these three genes in East Asian individuals with extremely high levels of HDL-C.

In the present study, 31 and 100% of the subjects possessed rare or common variants, respectively, of the three genes. These rates are higher than the values for rare or common variants (11 and 19% respectively) reported in a previous study performed in Canada^7^. However, while the present study included only individuals with HDL-C values > 100 mg/dL, the aforementioned study included those with HDL-C values of 54–70 mg/dL. Thus, phenotypic differences between the study populations may be related to the discrepancy in variant frequencies. Furthermore, another previous study using targeted next-generation sequencing reported that 5.2% of participants with extremely high levels of HDL-C (mean HDL-C: 96 mg/dL) possessed variants that were either causing or probably causing^12^. In contrast, the majority of the subjects in the present study possessed causing variants. The underlying reason for this difference remains to be clarified. In the Canadian study, large-effect variants were most frequent in *LIPC* in individuals with extremely high levels of HDL-C. However, most rare variants in the current analysis were variants of *CETP*.

It has been previously reported that homozygous loss-of-function mutations in *CETP* are associated with 80–100% higher levels of HDL-C^13,14^. Moreover, common loss-of-function variants of this gene are related to 20–30% higher values of HDL-C^14^. To date, a few rare *CETP* gene defects have been identified, such as the D442G variant in exon 15^15^ and G181X in exon 10^9^. According to a study using *in vitro* point mutagenesis, mutations of the hydrophobic amino acids at positions 454–475 are associated with a reduction in cholesteryl ester transfer activity^10^. Notably, two of the variants found in the present study were located at position 399. One of the most studied *CETP* polymorphisms is Taq1B, which is known to affect HDL-C levels in homozygous carriers^16^. We also found a common variant at position 362 of *CETP* that was present in most study subjects, though it was predicted to be benign. In addition, while we identified two novel variants of *CETP* in our subjects, further study is needed to determine their functional and clinical relevance.

With regard to *LIPC*, the S267F and 480C > T variants have been reported to be associated with high HDL-C levels^17,18^. In addition, several common variants were reported to be correlated with high HDL-C levels^19,20^. In our study, only common variants were found in *LIPC*, and of these, only N215S was predicted to be causing. Accordingly, rare variants of *LIPC* seem to contribute very little to the phenotype of extremely high HDL-C in our population.

Recently, it has been shown that the P376L variant of *SCARB1* is associated with elevated HDL-C levels and risk of coronary heart disease^21^. This study analysed individuals with extremely high levels of HDL-C, with a mean of 107 mg/dL. Of 328 subjects, five subjects (1.5%) carried homozygous or heterozygous variants, and when three cohorts were combined, the prevalence was 1.8%. In this regard, we could assume that variants of *SCARB1* are very rare in the general population. In a prior study, c.889C > T (P297S), a causing variant, was discovered in one of 162 Caucasians with HDL-C levels above the 95^th^ percentile^22^. In another study, two out of 120 Caucasians (1.7%) with HDL-C levels above the 90^th^ percentile were found to have S112F (C588T) or T175A (A776T), which are both rare causing variants^23^. Conversely, in a large data of whole-genome sequenced Icelanders, three rare variants (combined allelic frequency of 0.2%), one low-frequency variant, and three common variants of *SCARB1* were found to be associated with elevated HDL-C^24^. Furthermore, an American study that analysed subjects with HDL-C levels in the highest and lowest deciles found three common and novel *SCARB1* variants associated with HDL-C levels^25^. In our study, we identified only the D249N variant of *SCARB1* in one of the 42 subjects (2.4%). These results suggest that *SCARB1* variants may be diversely distributed in various populations.

Although studies on genetic variants associated with CEC have been very limited^26^, variants of genes affecting HDL metabolism have been suggested as candidates^27^. In a previous study, eight individuals with variants of *CETP* or *LIPC* and HDL-C values ≥ 105 mg/dL exhibited elevated CEC values. This finding was reported to be the result of increased HDL2 and the enhanced intrinsic capacity of HDL3^28^. In a recent study, one *LIPC* and two *CETP* variants revealed associations with ATP transporter-dependent CEC. However, the significance disappeared after adjusting for HDL-C and triglyceride levels^29^. Conversely, CEC values were similar between carriers and non-carriers of the P376L variant of *SCARB1*^21^. Nevertheless, since the carriers of the P297S variant had low CEC values in spite of high HDL-C levels^22^, the relationship between *SCARB1* variants and CEC values appear to be inconsistent. In the current study, CEC was not associated with the numbers of variants of the target genes or the presence of any specific variant. Furthermore, while we compared individuals according to genotype, the phenotypes of our subjects—particularly their HDL-C values—were very similar, which could have influenced our results.

In our study, the c.A1196G (p.D399G; rs2303790) variant of *CETP* showed a tendency, although not significant, of lower VCAM-1 expression. In previous studies, this variant showed association with eye pathologies including retinal disease^30,31^. The relationship between this variant and cardiovascular disease has been inconsistent^30,31^. Interestingly, it has been reported that HDL from CETP deficient individuals could have differential impact on VCAM-1 inhibition^32^. However, further studies are needed to clarify our findings regarding the effect of this CETP variant on VCAM-1.

While our study provides important genetic information of extremely elevated levels of HDL-C in Koreans, our study has some potential limitations. First, we did not collect data on family history or acquire samples from the family members of our subjects. An analysis of variants using co-segregation might have provided additional insight into their functionality. Second, the prediction of the causality of variants using publicly available tools for the analyses can be imperfect, and this may be another limitation. Third, we attempted to identify relationships between the genetic variants and CEC. However, as previously mentioned, the phenotypes of our subjects were quite homogeneous, and thus it was likely more challenging to identify the differential effects of the variants. Finally, we could not include a control group with normal HDL-C levels in our study. Having such a control group might have provided clearer insights about the effect of variants identified in the study.

In conclusion, rare or common variants of *CETP* and common variants of *LIPC* were frequently found in the study population with extremely high levels of HDL-C, while *SCARB1* variants were very uncommon. The presence of the identified variants was not associated with CEC, ROS generation, and VCAM-1 expression in the subjects of the present study. Our results provide comprehensive data regarding the spectrum of genetic variants of three target genes in East Asians with this HDL-C phenotype.

## Methods

### Study population

The Institutional Review Board of Severance Hospital approved the methods of the present study and all subjects and/or their legal guardians provided written informed consent. All research was performed in accordance with relevant guidelines/regulations. Between November 2000 and March 2011, 13,545 subjects were enrolled in the Cardiovascular Genome Center Cohort, Yonsei University College of Medicine, Seoul, Korea. Individuals ≥18 years of age were recruited into this cohort when they visited Severance Hospital for cardiovascular diseases, the control of risk factors, or health check-ups. Participants were interviewed regarding their medical history, after which they underwent a physical examination and laboratory evaluation. Of these individuals, 42 subjects whose HDL-C levels were >100 mg/dL were analysed. These subjects were not undergoing lipid-lowering therapy prior to enrolment in the current study. Pregnant women, individuals affected by cancer or thyroid, liver, or kidney disease, or patients undergoing pharmacotherapy that could affect lipid profiles (such as lipid-modifying agents, corticosteroids, or oral oestrogen) were excluded.

### Assessment of laboratory values, CEC, ROS, and VCAM-1

The levels of total cholesterol, triglyceride, HDL-C, and LDL-C were measured in all study participants. The participants fasted and avoided alcohol for at least 12 h prior to blood sampling. Samples were analysed within 4 h by a laboratory certified by the Korean Society of Laboratory Medicine. We analysed the potential relationships between the variants we sequenced here and CEC, a functional parameter of HDL. Assays for CEC, ROS, and VCAM-1 are described in the Supplementary Information.

### Targeted sequencing and analyses of variants

Three target genes were sequenced: *CETP* (MIM 118470), *LIPC* (MIM 151670), and *SCARB1* (MIM 601040). Genomic DNA was extracted from blood using the Qiagen Dneasy kit (Qiagen, Valencia, CA, USA). For mutation analyses, a panel for targeted DNA capture and sequencing was developed by Celemics, Inc. (Seoul, Korea). Targeted sequencing and variant analyses were performed as follows. DNA fragments that contained all coding exons and exon-intron junctions were enriched by solution-based hybridization capture, followed by sequencing using the Illumina HiSeq. 2000 platform (Illumina, Inc., San Diego, CA, USA). The quality of next-generation sequencing data, including coverage information, is presented in Supplementary Fig. S1.

An analysis of sequencing data was conducted using an in-house analysis pipeline as previously described^3^. Briefly, sequencing reads from the HiSeq. 2000 raw data were sorted by index and barcode sequences. Sorted FASTQ files were aligned to the hg19 reference genome using the Burrows-Wheeler Aligner (BWA; ver. 0.7.12) BWA-MEM algorithm. Output files in SAM format were converted into BAM files and sorted using SAMtools (ver. 1.1). Duplicate removal was performed with Picard tools (ver. 1.128) MarkDuplicates. Realignment around known indel sites and base quality score recalibration (BQSR) were conducted using GATK (v3.3.0) to create the final BAM files. Variants were called using the GATK v3.3.0 Unified Genotyper algorithm for loci with a sequencing depth greater than or equal to 50×. An analysis of splice site regions, including sufficient intronic bases, was performed using Human Splicing Finder.

The functional annotation of genetic variants was performed by ANNOVAR (ver. 2014-11-12). The predictions of the functional effects of single-nucleotide variants were acquired using SIFT, PolyPhen-2, and MutationTaster, and were matched against the Korean population exome data (n = 476) and a public database of variants (dsSNP 138, Exome Variant Server, and 1000 Genome Project SNP [April 2012 release] from both Asian and all-population databases). We then prioritized variants according to the following criteria: (1) variants that were reported to be disease-causing in the Human Gene Mutation Database, (2) disruptive variants (nonsense, splice-site [two nucleotides on either side of the intron/exon boundary], and frameshift) that were novel or rare, and (3) novel or rare missense variants that were predicted to be deleterious by any of the three prediction programs. Variants that met these criteria were validated by bidirectional Sanger sequencing of PCR amplicons. Databases used for confirming the identity and frequency of the variants included 1000 Genome Project, Exome Sequencing Project 6500, and gnomAD browser (http://gnomad.broadinstitute.org/). Variants with a minor allele frequency (MAF) of <1% were classified as rare, whereas those with a MAF of ≥5% according to public databases were classified as common.

### Statistical analyses

Continuous variables are presented as the mean ± standard deviation or median (interquartile range) and were compared using an independent *t*-test or Kruskal-Wallis test depending on the distribution of the data. Categorical variables were compared using a chi-square test or Fisher’s exact test. To analyse associations between genetic variants and the CEC of the test subjects, individuals were divided into four groups according to the quartile values of CEC. All statistical analyses were performed using R software version 3.5.0 (R Foundation for Statistical Computing, Vienna, Austria). For all analyses, differences with *p* < 0.05 were considered to be significant.

## Supplementary information

Supplementary information
