# Supplementary material for: CETP, LIPC, and SCARB1 variants in individuals with extremely high high-density lipoprotein-cholesterol levels
Source: Sci Rep. 2019 Jul 29;9:10915. doi: 10.1038/s41598-019-47456-2 (PMC6662756; doi:10.1038/s41598-019-47456-2)
Supplement: Supplementary file 1 — Supplementary information [file 41598_2019_47456_MOESM1_ESM.docx]

***CETP*, *LIPC*, and *SCARB1* variants in individuals with extremely high high-density lipoprotein-cholesterol levels**

Chan Joo Lee^a^, Mun Su Park^b^, Miso Kim^c^, Soo-jin Ann^c^, Jaeho Lee^c^, Sungha Park^a,c^, Seok-Min Kang^a,c^, Yangsoo Jang^a,c^, Ji Hyun Lee^d,e,*^, Sang-Hak Lee^a,c,*^

**Supplementary Information**

**Supplementary Methods**

**Assessment of CEC**

The CEC assay was performed as previously described^1^. Briefly, J774A1 cells (RRID: CVDL_0358) were plated and radiolabeled with 2 µCi of ^3^H-cholesterol/mL for 24 h. The cells were kindly provided by Yury I Miller at University of California, San Diego, USA. For the upregulation of adenosine triphosphate-binding cassette transporter subfamily member A1, the cells were incubated with medium containing 0.2% bovine serum albumin and 0.3 mM 8-(4-chlorophenylthio)-cyclic adenosine monophosphate for 6 h as previously described^2,3^. The medium was then changed to a medium containing 0.2% bovine serum albumin and the patient’s sample for 4 h^4,5^. The experiment was conducted by treating the cells with 2 µg/mL of an acyl-coenzyme A:cholesterol acyltransferase inhibitor. The cholesterol efflux proportion was calculated using the following formula: CEC (%) = {^3^H-cholesterol (µCi) in medium containing HDL/[^3^H-cholesterol (µCi) in medium containing HDL + ^3^H-cholesterol (µCi) in cells]} × 100. We subtracted background value from all sample values. The values were adjusted based on the efflux capacity of the pooled serum that was run in each plate. Each sample was run in duplicate.

**Assessment of reactive oxygen species (ROS)**

The generation of intracellular ROS was determined using dichlorodihydrofluorescein diacetate (CM-H2DCFDA, ThermoFisher Scientific, Waltham, MA, USA)^6,7^. J774 cells in 12-well plates (3 x 105 cells/well) were treated with subject’s sample for 24h. After staining with 5 μM CM-H2DCFDA in PBS for 45 min at 37°C, cells were incubated in the presence or absence of 100 μM hydroperoxide for 20 min. ROS generation was then determined with a flow cytometer. The mean fluorescence intensity was measured in 10,000 cells using the fluorescein isothiocyanate channel.

**Assessment of vascular cell adhesion molecule-1 (VCAM-1)**

The VCAM-1 expression was measured by western blotting^6,8^. Briefly, the human umbilical vein endothelial cells were grown and incubated with subject’s sample for 16 h at 37°C. After the cells were stimulated with 5 ng/mL of tumor necrosis factor-α , the cells were washed, and lysed in radioimmunoprecipitation assay buffer supplemented with protease inhibitor cocktail tablet (Roche Applied Science, Penzberg, Germany). Thereafter, the total protein concentration of cell lysate supernatant was determined. Then, 7 μg of protein was loaded and separated by running the 10% sodium dodecyl sulphate-polyacrylamide gel electrophoresis. The proteins were transferred from the gel to a nitrocellulose membrane and incubated with anti-VCAM-1 (Abcam, Cambridge, MA, USA) and mouse anti-β-actin antibodies (Santa Cruz Biotechnology, Inc., Santa Cruz, CA, USA). Protein bands were visualized using the SuperSignal West Pico Chemiluminescent substrate (ThermoFisher Scientific), and the band intensity was quantified using the ImageJ software (National Institute of Health, Bethesda, MD, USA). The VCAM-1 expression was normalized to the intensity of β-actin, and the levels in the cells treated with each subject’s sample were presented as percentages of untreated cells.

**Supplementary Figure S1.** NGS data statistics of targeted sequencing (A) The blue diamond indicates the average sequencing depth of the target region. (B) The blue histogram represents the coverage of the target region in each sample.


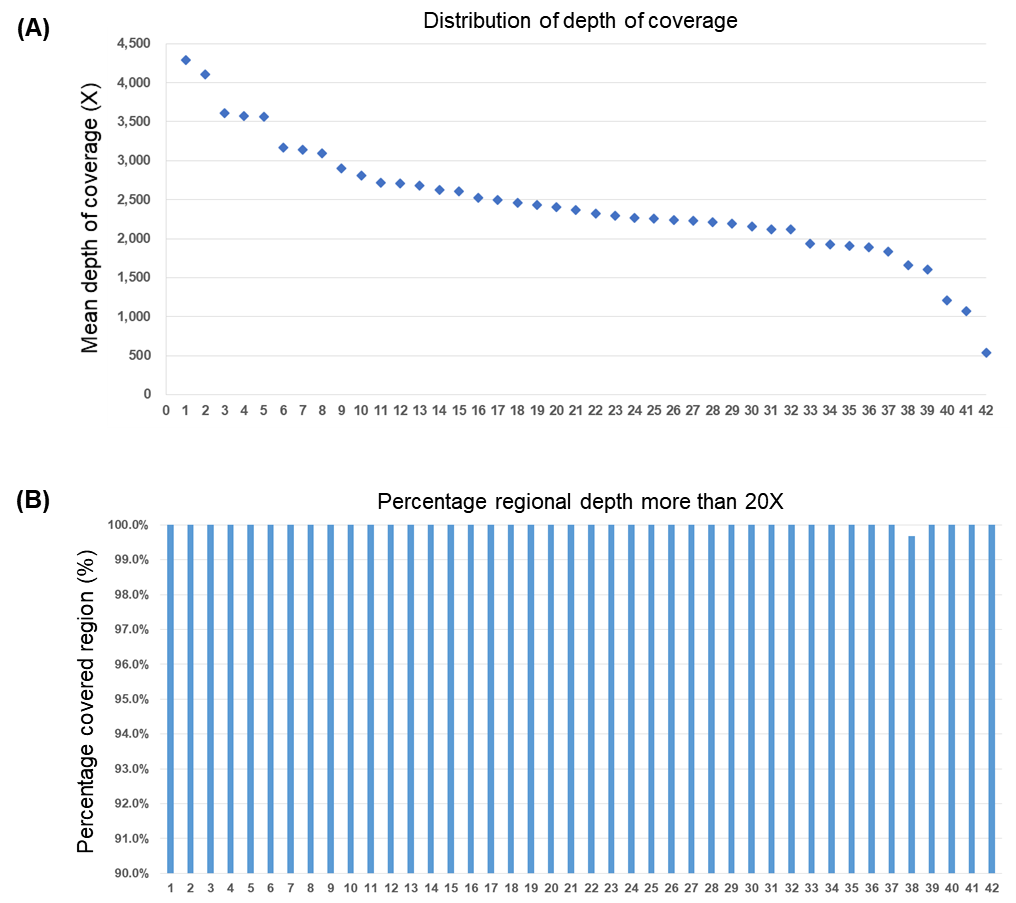


**Supplementary Table S1.** Summary of genetic variants of target genes identified in each individual

| Patients | Sex | Age | TC (mg/dL) | TG (mg/dL) | HDL-C  (mg/dL) | Number of variants | | | | | | | | |
| --- | --- | --- | --- | --- | --- | --- | --- | --- | --- | --- | --- | --- | --- | --- |
|  |  |  |  |  |  | *CETP* | | | *LIPC* | | | *SCARB1* | | |
|  |  |  |  |  |  | Rare | Common | Unknown | Rare | Common | Unknown | Rare | Common | Unknown |
| 1 | M | 43 | 273 | 48 | 162 | 0 | 1 | 0 | 0 | 3 | 0 | 0 | 0 | 0 |
| 2 | F | 53 | 202 | 68 | 140 | 0 | 1 | 0 | 0 | 3 | 0 | 0 | 0 | 0 |
| 3 | M | 43 | 233 | 113 | 128 | 1 | 1 | 0 | 0 | 3 | 0 | 0 | 0 | 0 |
| 4 | F | 25 | 309 | 63 | 117 | 0 | 1 | 0 | 0 | 2 | 0 | 0 | 0 | 0 |
| 5 | M | 52 | 272 | 76 | 113 | 0 | 0 | 0 | 0 | 2 | 0 | 0 | 0 | 0 |
| 6 | F | 37 | 196 | 45 | 111 | 0 | 1 | 0 | 0 | 2 | 0 | 0 | 0 | 0 |
| 7 | F | 45 | 230 | 27 | 110 | 0 | 1 | 0 | 0 | 2 | 0 | 0 | 0 | 0 |
| 8 | F | 56 | 327 | 42 | 107 | 0 | 1 | 0 | 0 | 2 | 0 | 0 | 0 | 0 |
| 9 | F | 52 | 232 | 68 | 107 | 0 | 1 | 0 | 0 | 2 | 0 | 0 | 0 | 0 |
| 10 | M | 10 | 226 | 50 | 105 | 0 | 1 | 0 | 0 | 2 | 0 | 0 | 0 | 0 |
| 11 | M | 35 | 182 | 77 | 104 | 0 | 1 | 0 | 0 | 3 | 0 | 0 | 0 | 0 |
| 12 | M | 52 | 172 | 143 | 103 | 0 | 1 | 0 | 0 | 2 | 0 | 0 | 0 | 0 |
| 13 | M | 59 | 210 | 55 | 101 | 0 | 1 | 0 | 0 | 3 | 0 | 0 | 0 | 0 |
| 14 | F | 62 | 204 | 142 | 100 | 1 | 1 | 0 | 0 | 3 | 0 | 0 | 0 | 0 |
| 15 | F | 55 | 275 | 78 | 100 | 0 | 1 | 0 | 0 | 2 | 0 | 0 | 0 | 0 |
| 16 | F | 55 | 256 | 51 | 100 | 0 | 1 | 0 | 0 | 3 | 0 | 0 | 0 | 0 |
| 17 | F | 55 | 199 | 70 | 103 | 1 | 1 | 0 | 0 | 3 | 0 | 0 | 0 | 0 |
| 18 | M | 73 | 255 | 96 | 145 | 0 | 1 | 0 | 0 | 3 | 0 | 0 | 0 | 0 |
| 19 | F | 73 | 221 | 53 | 118 | 0 | 1 | 1 | 0 | 2 | 0 | 0 | 0 | 0 |
| 20 | F | 50 | 218 | 42 | 115 | 1 | 1 | 0 | 0 | 2 | 0 | 0 | 0 | 0 |
| 21 | F | 56 | 199 | 78 | 113 | 0 | 1 | 0 | 0 | 3 | 0 | 0 | 0 | 0 |
| 22 | M | 70 | 164 | 42 | 112 | 1 | 1 | 0 | 0 | 2 | 0 | 0 | 0 | 0 |
| 23 | F | 44 | 244 | 52 | 111 | 0 | 1 | 0 | 0 | 2 | 0 | 0 | 0 | 0 |
| 24 | F | 64 | 164 | 48 | 111 | 0 | 1 | 1 | 0 | 2 | 0 | 0 | 0 | 0 |
| 25 | M | 66 | 191 | 43 | 111 | 0 | 1 | 0 | 0 | 3 | 0 | 0 | 0 | 0 |
| 26 | F | 58 | 201 | 58 | 110 | 1 | 1 | 0 | 0 | 2 | 0 | 0 | 0 | 0 |
| 27 | M | 65 | 165 | 62 | 110 | 0 | 1 | 0 | 0 | 2 | 0 | 0 | 0 | 0 |
| 28 | F | 64 | 224 | 58 | 109 | 1 | 1 | 0 | 0 | 3 | 0 | 1 | 0 | 0 |
| 29 | F | 52 | 196 | 58 | 109 | 0 | 0 | 0 | 0 | 2 | 0 | 0 | 0 | 0 |
| 30 | M | 73 | 171 | 113 | 108 | 1 | 1 | 0 | 0 | 2 | 0 | 0 | 0 | 0 |
| 31 | F | 59 | 261 | 67 | 106 | 0 | 1 | 0 | 0 | 2 | 0 | 0 | 0 | 0 |
| 32 | F | 45 | 187 | 65 | 105 | 0 | 1 | 0 | 0 | 3 | 0 | 0 | 0 | 0 |
| 33 | F | 62 | 279 | 120 | 105 | 1 | 1 | 0 | 0 | 2 | 0 | 0 | 0 | 0 |
| 34 | F | 51 | 225 | 76 | 104 | 0 | 1 | 0 | 0 | 3 | 0 | 0 | 0 | 0 |
| 35 | F | 52 | 197 | 44 | 103 | 1 | 1 | 0 | 0 | 2 | 0 | 0 | 0 | 0 |
| 36 | F | 51 | 217 | 62 | 103 | 0 | 0 | 0 | 0 | 2 | 0 | 0 | 0 | 0 |
| 37 | M | 70 | 231 | 122 | 102 | 1 | 1 | 0 | 0 | 2 | 0 | 0 | 0 | 0 |
| 38 | F | 61 | 213 | 87 | 102 | 0 | 0 | 0 | 0 | 2 | 0 | 0 | 0 | 0 |
| 39 | M | 52 | 182 | 52 | 102 | 0 | 1 | 0 | 0 | 3 | 0 | 0 | 0 | 0 |
| 40 | M | 49 | 217 | 151 | 101 | 1 | 1 | 0 | 0 | 2 | 0 | 0 | 0 | 0 |
| 41 | M | 63 | 212 | 114 | 100 | 1 | 1 | 0 | 0 | 3 | 0 | 0 | 0 | 0 |
| 42 | F | 59 | 202 | 57 | 100 | 0 | 1 | 0 | 0 | 2 | 0 | 0 | 0 | 0 |
| TC: total cholesterol; TG: triglyceride; HDL-C: high-density lipoprotein-cholesterol | | | | | | | | | | | | | | |

Supplementary Table S2. Association between the burden of variants and reactive oxygen species (ROS) generation

|  | Quartiles of ROS generation | | | | *p* |
| --- | --- | --- | --- | --- | --- |
|  | 1^st^ (n = 10) | 2^nd^ (n = 9) | 3^rd^ (n = 10) | 4^th^ (n = 10) |  |
| ROS production, AU | 5.6 ± 3.3 | 11.1 ± 0.8 | 12.7 ± 0.4 | 16.7 ± 3.4 | <0.001 |
| Numbers of variants in subjects of each quartile group |  |  |  |  |  |
| All target genes | 3 (2-4) | 3 (2-4) | 3 (3-4) | 4 (3-5) | 0.66 |
| *CETP* | 1 (1-1) | 1 (1-2) | 1 (1-2) | 1.5 (1-2) | 0.29 |
| *LIPC* | 2 (2-3) | 2 (1-2) | 2 (2-2) | 2 (2-3) | 0.27 |
| Numbers of variant carriers in each quartile group |  |  |  |  |  |
| *CETP* |  |  |  |  |  |
| c.A1196G (p.D399G) | 1 | 2 | 3 | 4 | 0.47 |
| c.G1195T (p.D399Y) | 0 | 1 | 0 | 0 | 0.33 |
| c.G1084A (p.V362I) | 8 | 9 | 9 | 8 | 0.51 |
| c.T974C (p.V325A) | 0 | 1 | 0 | 0 | 0.33 |
| c.G537A (p.W179X) | 0 | 0 | 1 | 0 | 0.40 |
| *LIPC* |  |  |  |  |  |
| c.C1068A (p.F356L) | 9 | 7 | 7 | 7 | 0.68 |
| c.A644G (p.N215S) | 10 | 9 | 10 | 10 | 0.99 |
| c.G283A (p.V95M) | 4 | 1 | 4 | 6 | 0.19 |
| *SCARB1* |  |  |  |  |  |
| c.G745A (p.D249N) | 0 | 1 | 0 | 0 | 0.33 |

Data are presented as mean + standard deviation or median (interquartile range) or number

AU: arbitrary unit

Supplementary Table S3. Association between the burden of variants and vascular cell adhesion molecule-1 (VCAM-1) expression

|  | Quartiles of VCAM-1 expression | | | | *p* |
| --- | --- | --- | --- | --- | --- |
|  | 1^st^ (n = 10) | 2^nd^ (n = 9) | 3^rd^ (n = 11) | 4^th^ (n = 9) |  |
| VCAM-1 expression^*^ | 4.1 ± 0.3 | 4.6 ± 0.1 | 5.0 ± 0.1 | 5.3 ± 0.1 | <0.001 |
| Numbers of variants in subjects of each quartile group |  |  |  |  |  |
| All target genes | 3.5 (2-4) | 4 (3-4) | 3 (2-3.5) | 4 (3-4) | 0.26 |
| *CETP* | 1.5 (1-2) | 2 (1-2) | 1 (1-1) | 1 (1-2) | 0.16 |
| *LIPC* | 2 (2-2) | 2 (2-3) | 2 (2-2) | 2 (2-3) | 0.54 |
| Numbers of variant carriers in each quartile group |  |  |  |  |  |
| *CETP* |  |  |  |  |  |
| c.A1196G (p.D399G) | 5 | 3 | 0 | 2 | 0.07 |
| c.G1195T (p.D399Y) | 0 | 0 | 0 | 1 | 0.33 |
| c.G1084A (p.V362I) | 8 | 9 | 9 | 8 | 0.55 |
| c.T974C (p.V325A) | 0 | 1 | 0 | 0 | 0.33 |
| c.G537A (p.W179X) | 0 | 1 | 0 | 0 | 0.33 |
| *LIPC* |  |  |  |  |  |
| c.C1068A (p.F356L) | 7 | 6 | 9 | 8 | 0.64 |
| c.A644G (p.N215S) | 10 | 9 | 11 | 9 | 0.37 |
| c.G283A (p.V95M) | 4 | 5 | 2 | 4 | 0.37 |
| *SCARB1* |  |  |  |  |  |
| c.G745A (p.D249N) | 0 | 0 | 0 | 1 | 0.33 |

Data are presented as mean + standard deviation or median (interquartile range)

*Log-transformed percentage of expression relative to the negative control group

Supplementary Table S4. Cholesterol efflux capacity (CEC), reactive oxygen species (ROS) production, and vascular cell adhesion molecule-1 (VCAM-1) expression of non-carriers and carriers of some variants of *CETP* and *LIPC*

| Genes | Variants |  | Carriers | Non-carriers | *p* |
| --- | --- | --- | --- | --- | --- |
| *CETP* | c.A1196G | Numbers | 11 | 31 |  |
|  |  | CEC, % | 24.8 ± 5.0 | 24.1 ± 5.6 | 0.73 |
|  |  | ROS, AU | 13.0 ± 3.7 | 11.0 ± 4.9 | 0.24 |
|  |  | VCAM-1 expression^*^ | 4.5 ± 0.5 | 4.8 ± 0.5 | 0.06 |
|  | c.G1084A | Numbers | 37 | 5 |  |
|  |  | CEC, % | 24.0 ± 5.2 | 26.0 ± 7.1 | 0.46 |
|  |  | ROS, AU | 11.6 ± 4.0 | 11.4 ± 8.9 | 0.97 |
|  |  | VCAM-1 expression^*^ | 4.7 ± 0.5 | 4.6 ± 0.8 | 0.64 |
| *LIPC* | c.G283A | Numbers | 17 | 25 |  |
|  |  | CEC, % | 25.0 ± 5.5 | 23.8 ± 5.4 | 0.48 |
|  |  | ROS, AU | 12.2 ± 5.3 | 11.1 ± 4.3 | 0.48 |
|  |  | VCAM-1 expression^*^ | 4.6 ± 0.5 | 4.8 ± 0.5 | 0.34 |

Data are presented as number or mean ± standard deviation.

AU: arbitrary unit

*Log-transformed percentage of expression relative to the negative control group

**References**

1. Cheon, E. J. *et al*. Novel association between CDKAL1 and cholesterol efflux capacity: replication after GWAS-based discovery. *Atherosclerosis*, **273,** 21–27 (2018).

2. Khera, A. V. *et al*. Cholesterol efflux capacity, high-density lipoprotein function, and atherosclerosis*. N. Engl. J. Med*. **364,** 127–135 (2011).

3. Saleheen, D. *et al.* Association of HDL cholesterol efflux capacity with incident coronary heart disease events: a prospective case-control study. *Lancet Diabetes Endocrinol*. **3,** 507–513 (2015).

4. [Haidar](https://www.ncbi.nlm.nih.gov/pubmed/?term=Haidar%20B%5BAuthor%5D&cauthor=true&cauthor_uid=12454270)b, B., [Denis](https://www.ncbi.nlm.nih.gov/pubmed/?term=Denis%20M%5BAuthor%5D&cauthor=true&cauthor_uid=12454270), M., [Krimbou](https://www.ncbi.nlm.nih.gov/pubmed/?term=Krimbou%20L%5BAuthor%5D&cauthor=true&cauthor_uid=12454270), L., [Marcil](https://www.ncbi.nlm.nih.gov/pubmed/?term=Marcil%20M%5BAuthor%5D&cauthor=true&cauthor_uid=12454270), M. & [Genest](https://www.ncbi.nlm.nih.gov/pubmed/?term=Genest%20J%20Jr%5BAuthor%5D&cauthor=true&cauthor_uid=12454270), J. Jr.. cAMP induces ABCA1 phosphorylation activity and promotes cholesterol efflux from fibroblasts. *J. Lipid Res*. **43,** 2087–2094 (2002).

5. Borja M. S. *et al*. [HDL-apolipoprotein A-I exchange is independently associated with cholesterol efflux capacity](https://www.ncbi.nlm.nih.gov/pubmed/26254308). *J. Lipid Res.* **56,** 2002–2009 (2015).

6. Lee. C. J. *et al*. Effect of two lipid-lowering strategies on high-density lipoprotein function and some HDL-related proteins: a randomized clinical trial. *Lipids. Health. Dis*. **16,** 49 (2017)

7. Jung. S. *et al*. Metabolic phenotyping of human atherosclerotic plaques: metabolic alterations and their biological relevance in plaque-containing aorta. Atherosclerosis. **269,** 21-28 (2018)

8. Speer. T. *et al*. Abnormal high-density lipoprotein induces endothelial dysfunction via activation of toll-like receptor-2. Immunity. **38,** 754-768 (2013)
